# Compensatory Feto-Placental Upregulation of the Nitric Oxide System during Fetal Growth Restriction

**DOI:** 10.1371/journal.pone.0045294

**Published:** 2012-09-27

**Authors:** Silvia Pisaneschi, Francesca A. L. Strigini, Angel M. Sanchez, Silvia Begliuomini, Elena Casarosa, Andrea Ripoli, Paolo Ghirri, Antonio Boldrini, Bruno Fink, Andrea R. Genazzani, Flavio Coceani, Tommaso Simoncini

**Affiliations:** 1 Division of Obstetrics and Gynecology, Department of Reproductive Medicine and Child Development, University of Pisa, Pisa, Italy; 2 Division of Neonatology, Department of Reproductive Medicine and Child Development, University of Pisa, Pisa, Italy; 3 Institute of Clinical Physiology, National Research Council, Pisa, Italy; 4 Noxygen Science Transfer and Diagnostics, Elzach, Germany; 5 Institute of Life Sciences, Scuola Superiore Sant’Anna, Pisa, Italy; VU University Medical Center, The Netherlands

## Abstract

**Background:**

Fetal Growth Restriction is often associated with a feto-placental vascular dysfunction conceivably involving endothelial cells. Our study aimed to verify this pathogenic role for feto-placental endothelial cells and, coincidentally, demonstrate any abnormality in the nitric oxide system.

**Methods:**

Prenatal assessment of feto-placental vascular function was combined with measurement of nitric oxide (in the form of S-nitrosohemoglobin) and its nitrite byproduct, and of the endogenous nitric oxide synthase inhibitor asymmetric dimethylarginine. Umbilical vein endothelial cells were also harvested to determine their gene profile. The study comprised term pregnancies with normal (n = 40) or small-for-gestational-age (n = 20) newborns, small-for-gestational-age preterm pregnancies (n = 15), and bi-chorial, bi-amniotic twin pregnancies with discordant fetal growth (n = 12).

**Results:**

Umbilical blood nitrite (p<0.001) and S-nitrosohemoglobin (p = 0.02) rose with fetal growth restriction while asymmetric dimethylarginine decreased (p = 0.003). Nitrite rise coincided with an abnormal Doppler profile from umbilical arteries. Fetal growth restriction umbilical vein endothelial cells produced more nitrite and also exhibited reciprocal changes in vasodilator (upwards) and vasoconstrictor (downwards) transcripts. Elevation in blood nitrite and S-nitrosohemoglobin persisted postnatally in the fetal growth restriction offspring.

**Conclusion:**

Fetal growth restriction is typified by increased nitric oxide production during pregnancy and after birth. This response is viewed as an adaptative event to sustain placental blood flow. However, its occurrence may modify the endothelial phenotype and may ultimately represent an element of risk for cardiovascular disease in adult life.

## Introduction

Fetal growth restriction (FGR) complicates up to 8% of pregnancies and carries high perinatal morbidity and mortality. [1] Infants born as small-for-gestational-age (SGA) also present a greater risk for cardiovascular disease during adulthood. [2] No definite explanation is available for the latter occurrence, although a deranged prenatal or perinatal programming of certain mechanisms [3], [4], [5] may be involved.

An alteration of the feto-placental circulation, reportedly involving the endothelium, plays a key part in the onset and progression of FGR. [6] Among the several agents controlling this vascular district, nitric oxide (NO) appears most important for its known role under normal conditions and as a target for pathological insults, [7] including possibly events that are identified with the development of FGR.^8,9^ However, besides being impaired by a variety of stimuli, NO may also have a compensatory function, and several situations in the adult document this possibility. [8].

Our objective was to study NO function in pregnancies complicated by FGR. The approach was comprehensive and included: (i) measurement of NO and its main metabolite, nitrite (NO_2_), along with the natural NO synthase inhibitor asymmetric dimethylarginine (ADMA); (ii) analysis of Doppler velocimetry in umbilical arteries; and (iii) assessment of gene profile in umbilical vein endothelial cells (HUVEC) collected at the time of delivery. Our ultimate aim was to define the nature of any linkage between NO and FGR and, coincidentally, provide a possible insight into the alleged negative impact of FGR on adult health.

**Figure 1:**
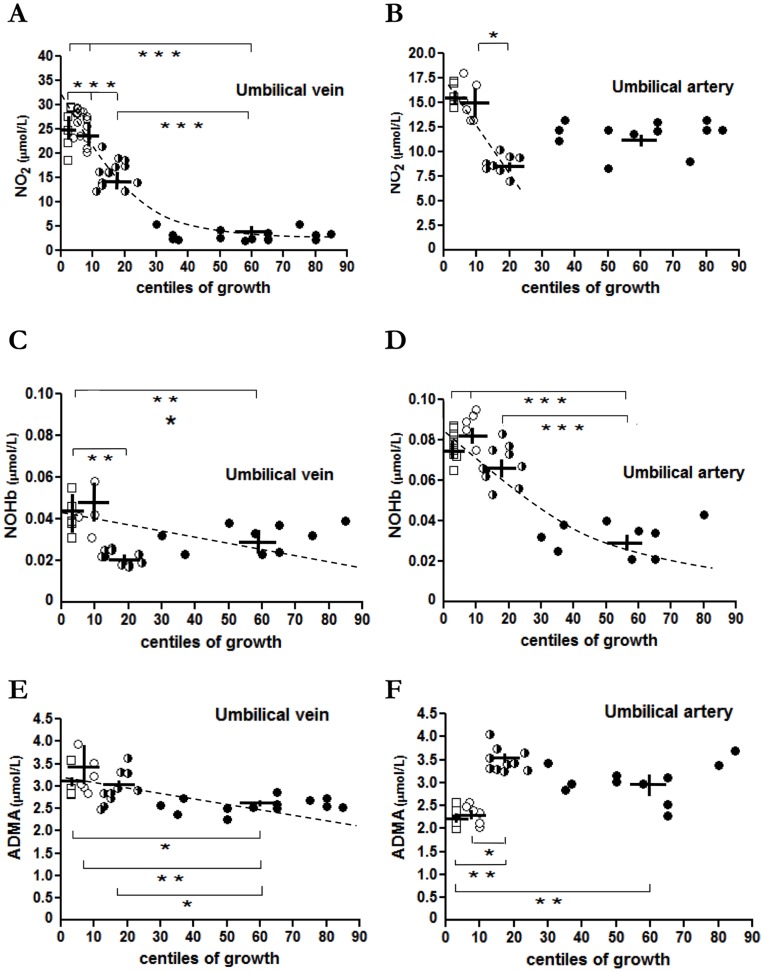
NO_2_, NOHb and ADMA levels in umbilical blood from pregnancies with normal or restricted fetal growth. Umbilical vein and artery levels of NO_2_ (**A, B**), NOHb (**C, D**) and ADMA (**E, F**) at different BW centiles: 75^th^–25^th^ (black circles); <25^th^–10^th^ (black and white circles); <10^th^–3^rd^ (white circles); <3^rd^ (white squares). Dashed line presents the exponential or linear relationship between these variable and BW. *p<0.05; **p<0.01; ***p<0.001.

## Materials and Methods

**Figure 2:**
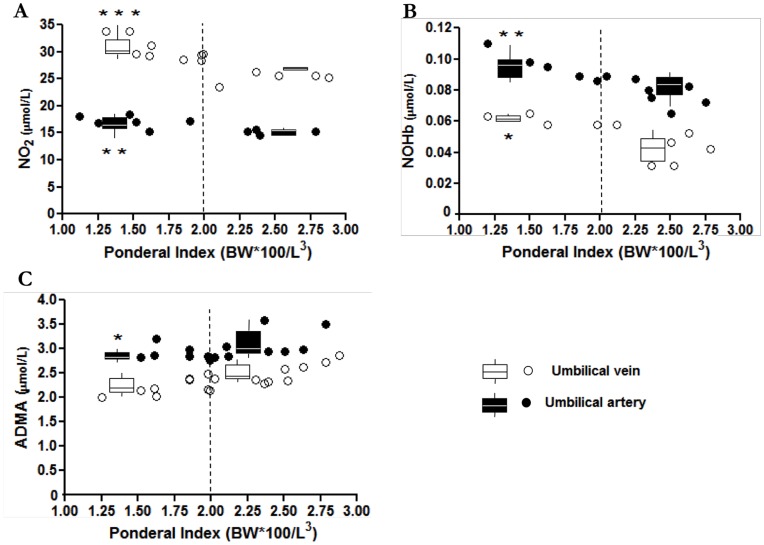
NO_2_, NOHb and ADMA levels in umbilical blood from pregnancies with symmetric or asymmetric fetal growth restriction. Umbilical vein and artery levels of NO_2_ (**A**), NOHb (**B**) and ADMA (**C**) in pregnancies with fetuses below 10th centile BW. Vertical dashed line separates groups with asymmetric (PInd <2) and symmetric (PInd >2) growth restriction. *p<0.05; **p<0.01; ***p<0.001 for <2 vs. >2 PInd groups.

### Study Groups

The study comprised three groups. Group 1: pregnancies being scheduled for umbilical Doppler velocimetry at 36 wk and yielding term infants with average (AGA) and small-for-gestational age (SGA; <2.5 kg at term or <10th percentile body weight) body weight (BW) (n = 60). Group 2: pregnancies with isolated preterm FGR (<10th percentile body weight; mean gestational age 34±1 wk, n = 15). Group 3: bi-chorionic, bi-amniotic twin pregnancies with offspring of discordant growth (mean gestational age 35±2 wk, n = 12 pregnancies). All patients included in the study were normotensive. A subset of neonates (n = 45, equally distributed among the groups 1 and 2) were subdivided according to BW in normal (75th–25th centile) (n = 15), at the limit of normality (<25th–10th centile) (n = 15) and in the SGA category (<10th centile, with <3rd centile being considered a severe SGA, n = 15). Mean ± SD of BW centiles for each group was as follows: 75th–25th centile: 52.82±14.45; <25th–10th centile: 17.13±4.23; <10th–3rd centile: 8.24±1.75; <3rd centile: 1.61±0.51. BW profile for the population is given in Fig. S1. SGA infants were further characterized by the Rohrer’s ponderal index (PInd) (weight in grams* 100/length in cm^3^). Average PInd with appropriate-for-gestational age infants at term is 2.4. [9] PInd decreases with FGR, because abdominal growth lags behind the increase in length (asymmetric growth). Thus, in its low range PInd can be used to characterize further the grade of FGR in newborns. Cord blood (artery and vein) was collected in all cases, while peripheral blood (heel sampling) was obtained from certain newborns at the time of delivery and 24 or 72 h afterwards. Blood being left after routine procedures was used for the latter purpose. Clinical characteristics of groups and demographic details are found in Tables S1, S2, and S3.

**Figure 3:**
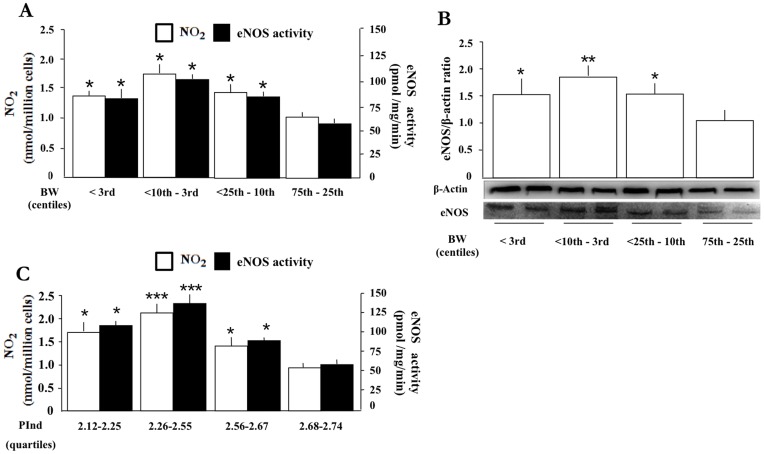
eNOS protein and activity in HUVEC from pregnancies with normal or restricted fetal growth. eNOS protein and activity, and NO_2_ content of incubate for HUVEC collected at different BW centiles (**A, B**). Values at (**C**) apply to different PInd grades within the 10^th^ centile BW group. *p<0.05; **p<0.01; ***p<0.001 vs. 75^th^–25^th^ centile (**A, B**) or 2.68–2.74 PInd (**C**).

### Ethics Statement

All participants provided written informed consent. Parents provided written approval for the procedures on the newborns, which did not add to the standard routine. The Ethics Committee of the University of Pisa approved protocol and procedures of the study.

### Doppler Velocimetric Analysis

Doppler ultrasound analysis was performed on the umbilical arteries with a Technos scanner (Esaote Biomedica, Genova, Italy) being equipped with color flow imaging, pulsed Doppler capability and convex probes (3.5–5 MHz for transabdominal examination). Records were stored for subsequent retrieval, and the same person (F.A.L.S.) was responsible for all the examinations.

Patients were studied in recumbent position after a 10-min rest. The result was labeled as abnormal in the case of absent or reversed end-diastolic flow. Similarly abnormal was considered any end-diastolic flow, with a peak-systolic/end-diastolic velocity ratio exceeding the mean value of the group by 2 SD.

**Figure 4:**
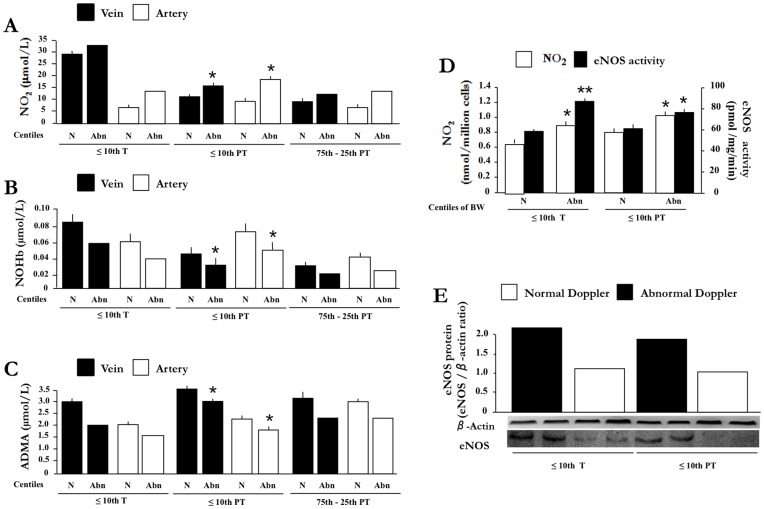
Prenatal umbilical artery Doppler velocimetry vs. NO function in fetuses with normal or restricted growth. Blood levels of NO_2_ (**A**), NOHb (**B**), and ADMA (**C)** for umbilical vein and artery with normal (N) vs. abnormal (Abn) Doppler velocimetry in term (T) or preterm (PT) pregnancies. Note that number of patients is low for certain groups (n = 1 or 2; see columns without SD bar), while n = 3–18 for the remainder. HUVEC from pregnancies with normal vs. abnormal Doppler: eNOS activity with attendant NO_2_ formation (**D**) (n = 3–4) and eNOS protein expression (**E**) (n = 2). *p<0.05; **p<0.01 vs. normal Doppler.

### Cell Culture

HUVEC from a subset of patients (n = 36, equally distributed among the indicated groups) were isolated at the time of delivery and were grown in culture according to a previous protocol [10] for measurement of endothelial nitric oxide synthase (eNOS) protein and activity. Miniarray analysis was performed on the same population of cells.

### Analytical Procedures

NO content of blood was assessed by measuring NO_2_ [10] and nitroso-hemoglobin (NOHb). The latter assay was carried out with the X-band electron paramagnetic resonance spectrometer ALEXIS (Bruker Biospin, Karlsruhe, Germany) in a high sensitivity microwave cavity. [11] Whole blood with known concentrations of NO_2_ (1–10 µM) and Na_2_S_2_O_4_ (20 mM) served as a reference. [12] ADMA was assayed in the same samples with a commercial kit (Pantec, Torino, Italy). eNOS activity of HUVEC was derived from the degree of arginine-to-citrulline conversion, [10] and NO_2_ was measured in parallel in the cell incubate.

**Figure 5:**
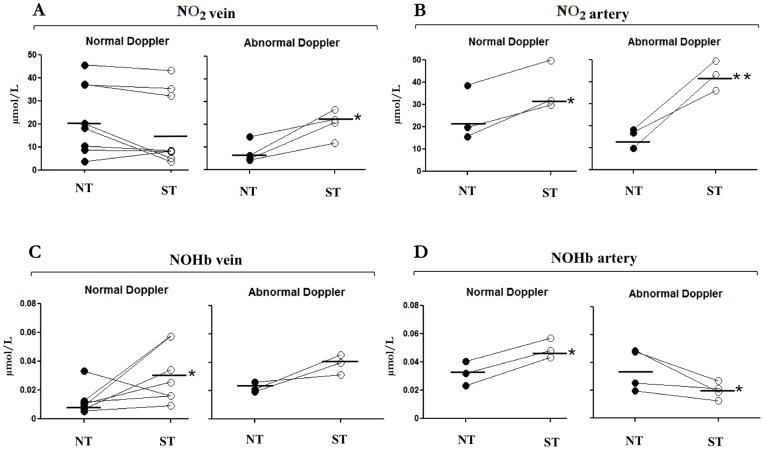
NO_2_ and NOHb levels in umbilical blood from twins with discordant intrauterine growth. NO_2_ (**A, B**) and NOHb (**C, D**) in umbilical vein and artery blood. NT: twin with normal growth; ST: twin with restricted growth. Note that points for each set of twins are connected by a line. *p<0.05; **p<0.01 vs. normal twin.

### Western Immunoblotting

HUVEC lysates were separated by SDS-PAGE and then subjected to immunoblotting for eNOS. Briefly, the primary antibody (rabbit anti-human eNOS, 1∶1000; Transduction Laboratories, Lexington, KY) was incubated with the blotted membranes overnight at 4°C and blots were subsequently hybridized with a secondary antibody (goat anti-rabbit, 1∶4000; Transduction Laboratories). β-Actin (Santa Cruz Biotechnology, Santa Cruz, CA) served as reference. The resulting chemiluminescent signal was recorded with a quantitative digital imaging system capable of recognizing any saturation of the input (Quantity One; BioRad, Hercules, CA). Results are given as the eNOS/β-actin ratio in arbitrary units (control = 1), with conditions ensuring the reading of signals over a linear range.

### Miniarray Analysis

Total RNA was extracted from HUVEC with the guanidine thiocianate-phenol-chloroform mixture, and hybridization of an aliquot (4 µg) was performed with Human Endothelial Cell Biology Oligo GEArrays (Superarray Biosciences, Frederick, USA). Singleton pregnancies being scheduled for umbilical Doppler velocimetry at 36 wk and yielding term infants with average (AGA) body weight were studied comparatively with isolated severe FGR singleton pregnancies (n = 6 per group). A total of 114 genes were examined (Table S4), all being selected for their general relevance to endothelial function. Gene expression was measured by chemiluminescence, taking care to ensure the same exposure for each membrane. Quantitative analysis was obtained with a digital system excluding any saturation (Quantity One; BioRad). The signal for each gene was measured against housekeeping genes incorporated into the system, as recommended by the manufacturer. Background noise was also subtracted in the process. A 5-fold change of expression in either direction was taken as the limit for acceptance of data.

**Figure 6:**
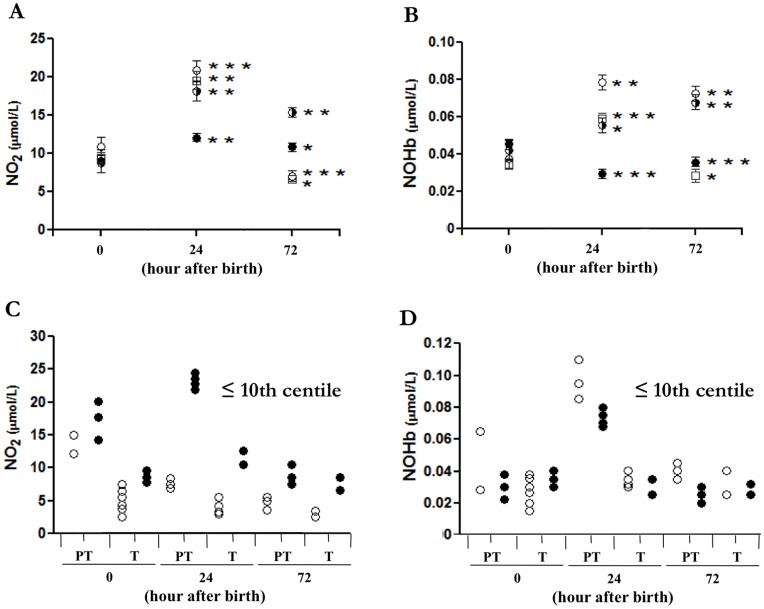
Time-related change in peripheral blood levels of NO_2_ and NOHb in AGA and SGA newborns. Blood levels of NO_2_ (**A, C**) and NOHb (**B, D**) in relation to BW centile (**A, B**) and prenatal Doppler finding (**C, D**) at different time intervals after birth. BW centile: 75^th^–25^th^ (black circles); <25^th^–10^th^ (black and white circles); <10^th^–3^rd^ (white circles); <3^rd^ (white squares). Doppler finding for term (T) and preterm (PT) pregnancy: normal (white circles) and abnormal (black circles) with each point representing a single patient. *p<0.05; **p<0.01; ***p<0.001 for 24- and 72- h intervals relative to time zero. Note that, in the aggregate, NO_2_ and NOHb values for 75^th^–25^th^ centile are significantly different from the other groups (p<0.001) at the 24- and 72-h time intervals, the only exception being the NOHb value for the <3^rd^ centile at the 72-h mark (p = 0.068).

### Statistics

Data are presented as the mean ± SD. Comparisons were made using Student’s t-test or ANOVA followed by the Tukey test. Regression analysis was performed with the best fitting for a linear or exponential relationship. Differences are considered significant with p<0.05.

## Results

### NO2, NOHb and ADMA in Umbilical Blood

Regression analysis showed a significant inverse relationship with exponential course between NO_2_ levels in the umbilical vein and growth of newborns (R^2^ = 0.809; p<0.001, Fig. 1A). NO_2_ elevation was particularly marked with BW below the 10th centile (Fig. 1A). A similar pattern emerged from umbilical artery sampling, but the magnitude of the NO_2_ change was smaller and a linear inverse relationship between NO_2_ and BW values was evident only from the 25^th^ centile downwards (R^2^ = 0.262; p = 0.018, Fig. 1B).

**Figure 7:**
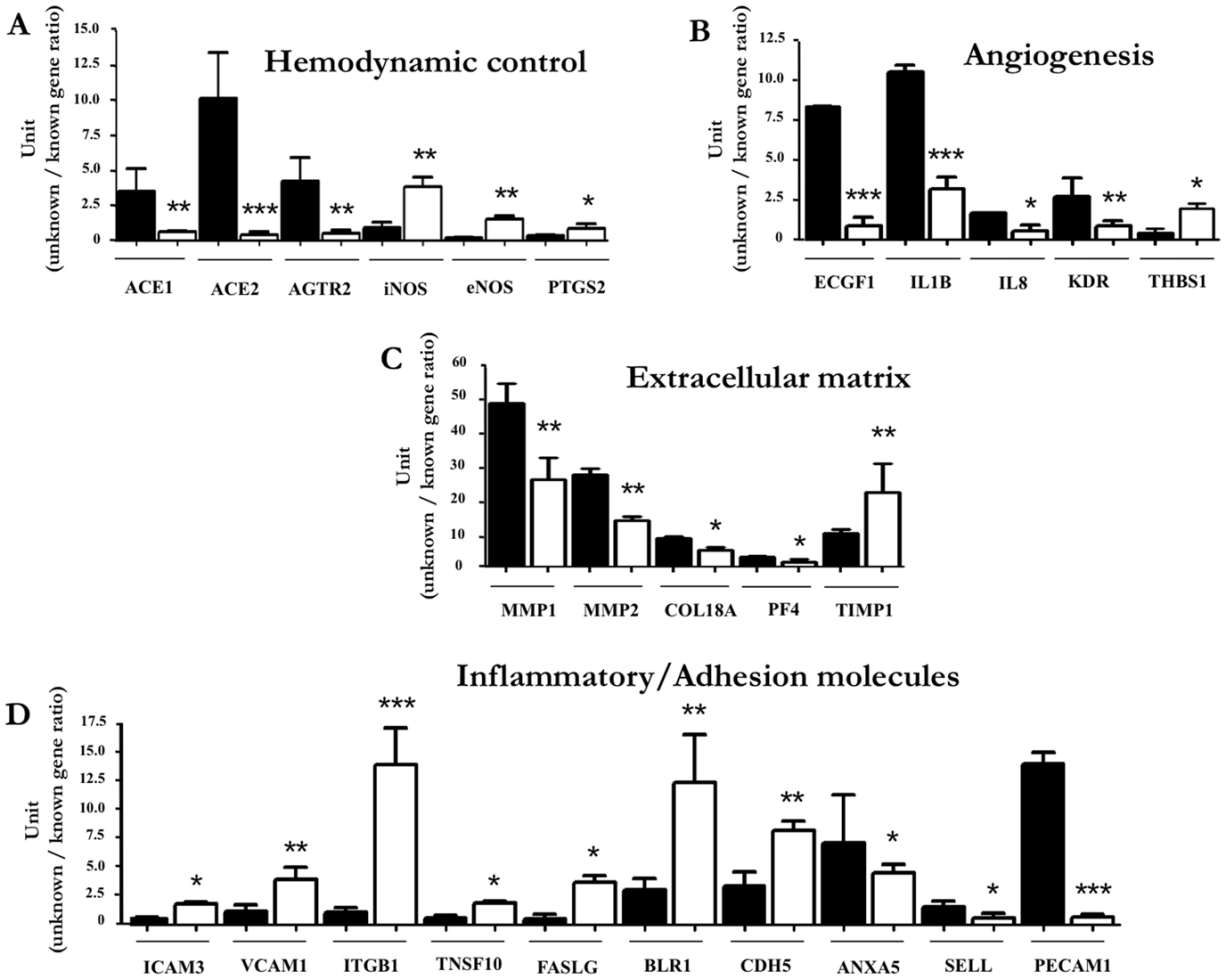
Gene profile in HUVEC from SGA vs. AGA pregnancies. Comparison of gene expression at 75^th^-25^th^ (black squares) vs. <3^rd^ (white squares) BW centile. Figure reports transcripts being affected by fetal growth restriction that relate to hemodynamic control (**A**), angiogenesis (**B**), extracellular matrix turnover (**C**) and inflammatory/adhesion process (**D**)**.** Values are expressed as the intensity ratio between test and reference genes (for details, see Methods). Note that the total gene cohort under examination is given in Table S4. *p<0.05; **p<0.01; ***p<0.001 for <3^rd^ vs. 75^th^–25^th^ BW centile.

NOHb was also elevated in umbilical blood of FGR pregnancies, regardless of whether it originated from the artery or the vein (Figg. 1C and 1D). However, at variance from NO_2_, the inverse relationship linking NOHb with BW was linear with the vein (R^2^ = 0.169; p = 0.024, Fig. 1C) and exponential with the artery (R^2^ = 0.705; p<0.001, Fig. 1D).

Contrary to NO_2_ and NOHb, changes in ADMA levels were less consistent and varied in sign depending on vessel and degree of FGR. As shown in Fig. 1E, findings with the umbilical vein paralleled those for NO-related variables, and a linear inverse relationship linked ADMA content with BW (R^2^ = 0.226; p = 0.003). No such relationship was noted instead with the umbilical artery (Fig. 1F), and ADMA was either decreased (<10^th^ centile) or marginally increased (<25^th^ –10^th^ centile).

A separate analysis was carried out with SGA pregnancies of BW below the 10^th^ centile, where levels of compounds in umbilical vein and artery were correlated with the PInd of infants. For this purpose, a PInd of 2 was taken as the dividing limit between symmetric and asymmetric growth groups (see Methods). As shown in Fig. 2, pregnancies with PInd below 2 had significantly higher amounts of NO_2_ and NOHb in both vessels. Conversely, under the same conditions ADMA level was lower only in the umbilical artery.

### eNOS Function in HUVEC

eNOS protein expression and activity and NO_2_ release were significantly higher in HUVEC from the 25th centile BW group downwards (Fig. 3A and 3B). Furthermore, the same pattern was seen when relating these variables to the PInd of newborns with BW below the 10^th^ centile. Fig. 3C documents this point, even though the subset of pregnancies was distributed over a relatively narrow range compared to that considered in Fig. 2.

### Umbilical Artery Doppler Analysis vs. NO Markers in Umbilical Blood and Cells from Pregnancies with Normal or Restricted Fetal Growth

A subset of the patients, including preterm and term pregnancies with FGR, also had a Doppler velocimetric analysis of the umbilical arteries. Limited to this group, NO_2_ levels in umbilical vein and artery blood were increased with an abnormal Doppler profile (Fig. 4A), while in contrast NOHb and ADMA levels were decreased (Fig. 4B and 4C). This finding appeared to be independent of fetal growth status or gestational age. In addition, eNOS protein and activity in HUVEC, with the attendant NO_2_ in the incubate, were elevated when dealing with SGA pregnancies with Doppler derangement (Fig. 4D and 4E).

### NO_2_ and NOHb in Umbilical Blood from Twins with Discordant Intrauterine Growth

Umbilical vein NO_2_ did not differ between normal (NT) and small (ST) twin when Doppler analysis was normal (Fig. 5A), while in contrast the compound was elevated in the umbilical artery of the small twin (Fig. 5B). No difference between the two vessels was noted with an abnormal Doppler, and NO_2_ levels were consistently higher in the SGA infant of the pair (Fig. 5A and 5B).

NOHb, on the other hand, was elevated in both umbilical vessels of the SGA infant even in the absence of any anomaly in the velocimetric analysis (Fig. 5C and 5D). However, when SGA was associated with an abnormal Doppler, NOHb abated in the umbilical artery (Fig. 5D) and tended to rise in the vein (Fig. 5C). Blood levels of ADMA in either vessel did not differ, regardless of BW and the state of umbilical artery blood flow (data not shown).

### Blood NO_2_ and NOHb in Newborns with Normal or Restricted Growth

As shown in Fig. 6 (A, B), newborns below the 25th centile presented a marked increase in blood NO_2_ and NOHb at 24 h of age which, in certain cases, was maintained up to the 72-h. Conversely, a modest elevation, only for (NO_2_), or no elevation at all was seen in the normal offspring. Furthermore, infants with an abnormal Doppler profile tended to have higher levels of blood NO with both term and preterm pregnancies (Fig. 6C). No such correlation was noted in the case of NOHb (Fig. 6D).

### Gene Profile in HUVEC from Fetuses with Normal or Restricted Growth

A group of 26 transcripts, out of a total of 114, changed their expression in either direction in HUVEC from SGA vs. AGA pregnancies (Fig. 7A–D). In particular, lesser expression was observed for angiotensin I converting enzyme 1 and 2 (ACE1 and ACE2) and angiotensin II receptor type 2 (AGTR2), while NOS enzymes and prostaglandin endoperoxide synthase-2 (PTGS2) were increased (Fig. 7A). SGA cells also presented a down-regulation of pro-angiogenetic factors, such as endothelial cell growth factor-1 (ECGF1), interleukin-1β (IL1B), interleukin-8 (IL8) and VEGF receptor (KDR) (Fig. 7B). Conversely, the anti-angiogenetic factor thrombospondin-1 (THBS1) was increased (Fig. 7B). Metalloproteinases (MMP) 1 and 2, collagen type XVIII alpha 1 (COL18A) and platelet factor 4 (PF4) were down-regulated, while the tissue inhibitor of metalloproteinase-1 (TIMP1) was up-regulated (Fig. 7C). Lastly, HUVEC from SGA pregnancies exhibited a differential pattern for inflammatory/adhesion genes (Fig. 7D). Specifically, the up-regulation of intercellular adhesion molecule-3 (ICAM3), vascular cell adhesion molecule-1 (VCAM1), integrin-β1 (ITGB1), FAS ligand (FASLG) and VE-cadherin (CDH5) vis-à-vis the down-regulation of annexin A5 (ANXA5), L-selectin (SELL) and platelet/endothelial cell adhesion molecule-1 (PECAM1) (Fig. 7D).

## Discussion

Our study shows activation of the NO system in pregnancies with FGR, specifically when placental vascular resistance is impaired. Elevation of NO_2_ and NOHb is ascribed to enhanced eNOS activity, within and outside the placenta. This adaptive response presents distinctive features in HUVEC where appropriate changes are seen in genes controlling blood flow, angiogenesis, extracellular matrix remodeling and inflammation. Furthermore, a NO enhancement is also evident after birth. Hence, findings suggest the existence of a re-programming of the feto-placental vascular system, and of the fetus as a whole, in the face of adverse intrauterine conditions. Expectedly, its immediate purpose is to preserve an adequate placental blood flow. An open question, however, is whether this event translates into a conditioning process for SGA newborns and, as a corollary, into an element of risk later in life. A possibility in this respect is that, in line with evidence from other sites [13], [14], an extended activation of the NO system is followed by down-regulation, specifically of vascular eNOS. The relative hyperoxia of the neonate would favor any such occurrence through depletion of tetrahydrobiopterin (BH_4_) secondary to oxygen radical formation. [15] Recent data in the rat are also consistent with the idea of an altered hemodynamic control in the adult resulting from FGR-linked NO dysfunction. [16] If confirmed, this concept could open the way to new therapeutic strategies, based on BH_4_ supplementation and/or free radical scavenging, to improve the postnatal adaptation of SGA infants.

The source of NO_2_ in the feto-placental circulation of FGR pregnancies remains to be determined. Pregnancies complicated by FGR show increased eNOS expression in syncytiotrophoblast cells. [17] Our finding of greater eNOS and iNOS expression in HUVEC confirms the importance of any up-regulation of NOS transcript(s) in the adaptation of the feto-placental vascular district to FGR. One should mention, however, that a reduction of eNOS activity has also been reported, [18] hence highlighting how this enzyme may be conditioned by a host of factors with opposing effects. A related question is whether agents that inhibit eNOS, such as ADMA, [19] are causally linked to FGR, in analogy to observations made with pre-eclampsia. [20] We found that, in the case of FGR, ADMA levels are decreased in the umbilical artery, while the opposite occurs in the vein. This finding accords with the idea of the fetus being able to reset its NO system upwards, and this event, along with a decreased production of ADMA, is conceivably crucial in preserving placental vascular flow. Any such resetting also applies to pregnancies with a severely growth-restricted fetus, where the decreasing ponderal index coincides with higher amounts of NO_2_ and NOHb in the umbilical vein. Indeed, this particular relationship is also evident after birth, since infants belonging to the low-centile groups (<25^th^ centile) present a more active NO system compared to those within a normal range (75^th^–25^th^ centile).

Significantly, we identified erythrocyte-based NOHb as a potential source for the elevated NO in FGR, even in conditions of altered feto-placental resistance. Methodologically, the measurement of NOHb has been introduced as a suitable way to monitor circulating NO in animals and humans. [12] Our report highlights how this novel technique can be exploited to assess NO function during pregnancy, with the attendant possibility of better defining clinical conditions such as an altered feto-placental vascular resistance or FGR. The existence, in fact, of a good matching between NO_2_/NOHb levels in the feto-placental circulation and indices of vascular resistance/flow provides the basis for any such approach.

NO function in FGR is sustained, in part, by an enhanced eNOS activity in feto-placental endothelial cells. Our analysis suggests the occurrence of a reset in endothelial function, being directed to the maintenance of blood flow in umbilical vessels. A qualifying event in this process is the reciprocal variation in eNOS (upwards) and angiotensin-converting enzyme/angiotensin receptor (downwards) expression. Coincidentally, HUVEC from SGA pregnancies manifest a gene expression pattern that would be unfavorable for angiogenesis and vascular remodeling. Some of these gene products – VEGF, VCAM-1 and ICAM-1– have previously been implicated in FGR or pre-eclampsia and have also been assigned predictive value for these conditions. [21], [22], [23] The overall picture emerging from these data is that feto-placental endothelial cells play a complex role in the pathophysiology of FGR, possibly by contributing to processes that limit the development of the placenta and its responsiveness to hypoxia.

Demonstration of a differential relationship between BW and umbilical blood levels of NO_2_ and NOHb in twins with discordant growth further supports a prime role of the feto-placental vasculature, and the fetus as a whole, in the natural course of FGR. Maternal factors are, in fact, common to the two situations and cannot account for the observed differences.

### Conclusion

In conclusion, we find that FGR pregnancies present an adjustment in the NO system being characterized by elevation of NO_2_ and NOHb and reduction of ADMA. Endothelial cells of the feto-placental circulation are part of this response through a phenotypic change that attests to growth restriction and hemodynamic dysfunction. Assessment of NO function in the clinical setting could become important for the prevention and management of FGR. Furthermore, if future studies were to confirm that changes in the NO system in SGA infants are linked to increased susceptibility to cardiovascular disorders later in life, new strategies may ensue for identification of individuals at risk and introduction of preventive measures.

## Supporting Information

Figure S1 **Distribution of body weight at birth and body weight for gestational age in the study population. (A)** BW distribution across centile groups. **(B)** BW for each centile group at different gestation ages. Values are means ± SD.
(TIFF)

Table S1 **Clinical characteristics of groups.**
(DOCX)

Table S2 **Clinical characteristics of twin pregnancies.**
(DOCX)

Table S3 **Demographic characteristics of the mothers.**
(DOCX)

Table S4 **Listing of genes under examination.**
(DOCX)
