# Supplementary figures and images for: Compensatory Feto-Placental Upregulation of the Nitric Oxide System during Fetal Growth Restriction
Source: PLoS One. 2012 Sep 27;7(9):e45294. doi: 10.1371/journal.pone.0045294 (PMC3459972; doi:10.1371/journal.pone.0045294)

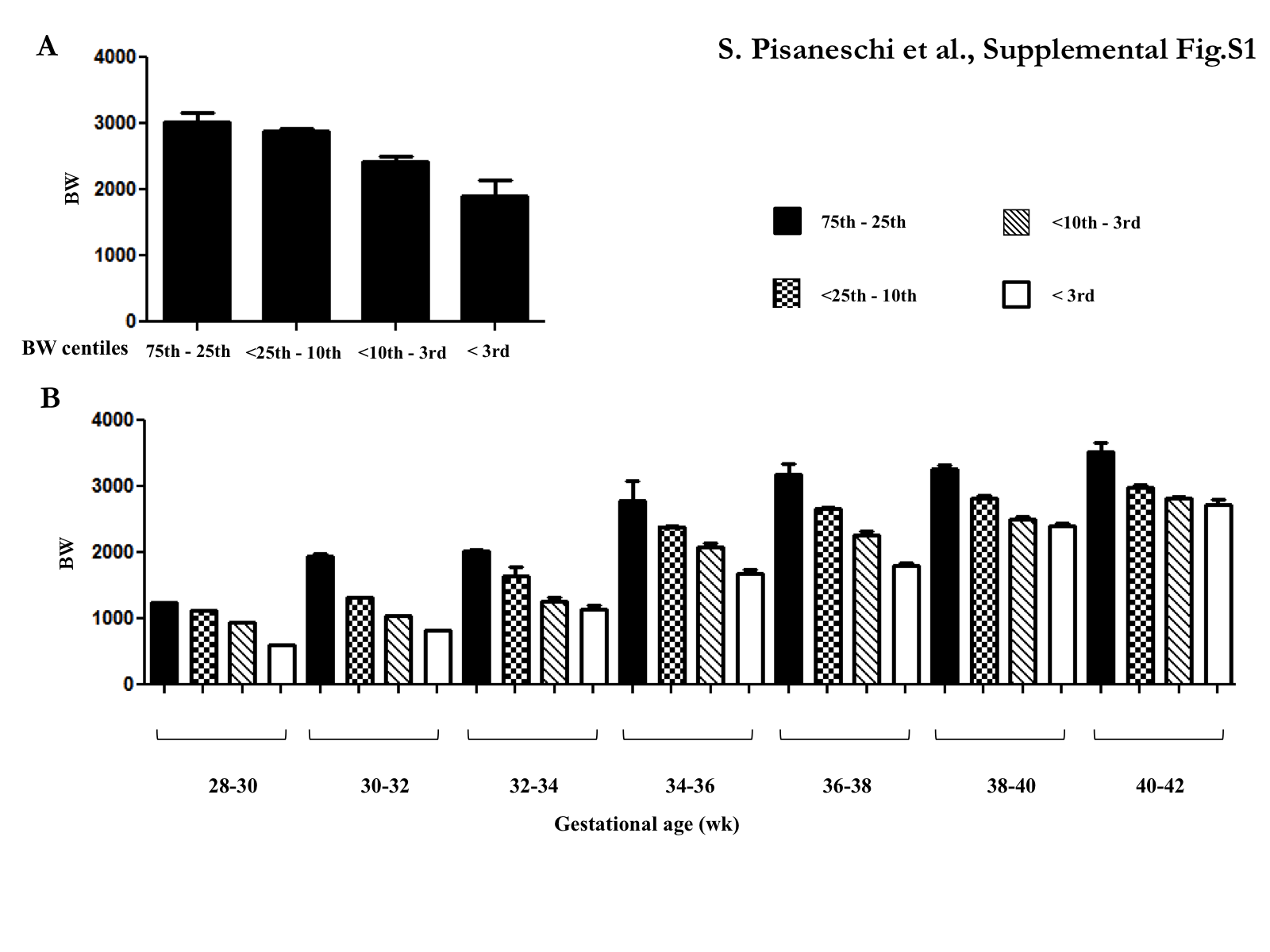

Supplement: Figure S1 — Distribution of body weight at birth and body weight for gestational age in the study population. (A) BW distribution across centile groups. (B) BW for each centile group at different gestation ages. Values are means ± SD. (TIFF) [file pone.0045294.s001.tif]
