# Supplementary material for: Compensatory Feto-Placental Upregulation of the Nitric Oxide System during Fetal Growth Restriction
Source: PLoS One. 2012 Sep 27;7(9):e45294. doi: 10.1371/journal.pone.0045294 (PMC3459972; doi:10.1371/journal.pone.0045294)
Supplement: Table S1 — Clinical characteristics of groups. (DOCX) [file pone.0045294.s002.docx]

| **Supporting Table 1. Clinical characteristics of groups** | | | | | | | | | | | | | |
| --- | --- | --- | --- | --- | --- | --- | --- | --- | --- | --- | --- | --- | --- |
|  | **75th-25th**  **(n = 25)** | | | | | **<25th-10th**  **(n = 20)** | | **< 10th centile**  **(n = 35)** | | | | | |
| **Doppler velocimetry** | Abnormal  (n = 1) | | Normal  (n = 4) | Normal  (n = 10) | Normal  (n = 10) | Normal  (n = 20) | | Abnormal  (n = 7) | | Normal  (n = 28) | | | |
| **Delivery** ^a^ | Cesarean  section | | | Cesarean section | Spontaneous delivery | Cesarean section  (n = 10) | Spontaneous delivery  (n = 10) | Cesarean  section | | Cesarean section  (n = 5) | Spontaneous delivery  (n = 5) | Cesarean section  (n = 9) | Spontaneous delivery  (n = 9) |
| **Gestational age** **(wk)** ^b^ | 29 | 32 ± 1 | | 38 ± 2 | 39± 1 | 39 ± 1 | 39 ± 1 | 31 ± 2 (n = 5) | 38  (n = 2) | 36 ± 1 | 35 ± 1 | 39 ± 1 | 39 ± 1 |
| **Male**  **Female** | 13  12 | | | | | 11  9 | | 18  17 | | | | | |
| ^a^ Twin pregnancies were delivered by cesarean section.  ^b^ Values are means ± SD. | | | | | | | | | | | | | |
