# Supplementary material for: Compensatory Feto-Placental Upregulation of the Nitric Oxide System during Fetal Growth Restriction
Source: PLoS One. 2012 Sep 27;7(9):e45294. doi: 10.1371/journal.pone.0045294 (PMC3459972; doi:10.1371/journal.pone.0045294)
Supplement: Table S2 — Clinical characteristics of twin pregnancies. (DOCX) [file pone.0045294.s003.docx]

| **Supporting Table 2. Clinical characteristics of twin pregnancies** | | | | |
| --- | --- | --- | --- | --- |
|  | **Normal Doppler velocimetry** | | **Abnormal Doppler velocimetry** | |
|  | **NORMAL TWIN**  **(n = 7)** | **SMALL TWIN**  **(n = 7)** | **NORMAL TWIN**  **(n = 5)** | **SMALL TWIN**  **(n = 5)** |
| **Gestational age** **(wk)** ^a^ | 35 ± 3 | | 33 ± 4 | |
| **Birth weight (g)** ^a^ | 2535 ± 79 | 2233 ± 57 | 1781 ± 26 | 1436 ± 26 |
| **BW centile** ^a^ | 23 ± 3 | 5 ± 1 | 26 ± 4 | 5 ± 4 |
| ^a^ Data are means ± SD. | | | | |
