# Supplementary material for: Compensatory Feto-Placental Upregulation of the Nitric Oxide System during Fetal Growth Restriction
Source: PLoS One. 2012 Sep 27;7(9):e45294. doi: 10.1371/journal.pone.0045294 (PMC3459972; doi:10.1371/journal.pone.0045294)
Supplement: Table S3 — Demographic characteristics of the mothers. (DOCX) [file pone.0045294.s004.docx]

| **Supporting Table 3. Demographic characteristics of the mothers** | | | | | |
| --- | --- | --- | --- | --- | --- |
|  |  | **75th-25th BW centile**  **(n = 25)** | **<25th-10th BW centile**  **(n = 20)** | **< 10th-3rd BW centile**  **(n = 30)** | **< 3rd BW centile**  **(n = 5)** |
| **Parity** | nullipara | 16/25 (64%) | 15/20 (75%) | 22/30 (73%) | 4/5 (80%) |
|  | primipara | 8/25 (32%) | 4/20 (20%) | 7/30 (23%) | 1/5 (20%) |
|  | pluripara | 1/25 (4%) | 1/20 (5%) | 1/30 (4%) | --- |
| **Maternal weight gain (kg)** ^a^ | | 13.6 ± 2.3 | 11.0 ± 2.4 * | 8.8 ± 1.0 * * | 6.6 ± 0.8 * * * |
| **Maternal age** **(yr)** ^a^ | | 34 ± 4 | 30 ± 4 | 32 ± 5 | 36 ± 2 |
| **Employment status** | yes | 17/25 (68%) | 13/20 (65%) | 17/30 (56%) | 1/5 (20%) |
|  | no | 8/25 (32%) | 7/20 (35%) | 13/30 (44%) | 4/5 (80%) |
| **Ethnic group** | Italian | 21/25 (84%) | 16/20 (80%) | 29/30 (95%) | 2/5 (40%) |
|  | Middle Eastern | 2/25 (8%) | 2/20 (10%) | 1/30 (5%) | 2/5 (40%) |
|  | African | 1/25 (4%) | 1/20 (5%) | ----- | 1/5 (20%) |
|  | Asian | 1/25 (4%) | 1/20 (5%) | ----- | ----- |
| ^a^ Values are means ± SD. * p < 0.05; * * p < 0.01; * * * p < 0.001 vs 75^th^-25^th^ BW centile | | | | | |
