# Supplementary material for: Compensatory Feto-Placental Upregulation of the Nitric Oxide System during Fetal Growth Restriction
Source: PLoS One. 2012 Sep 27;7(9):e45294. doi: 10.1371/journal.pone.0045294 (PMC3459972; doi:10.1371/journal.pone.0045294)
Supplement: Table S4 — Listing of genes under examination. (DOCX) [file pone.0045294.s005.docx]

| **Supporting Table 4. Listing of genes under examination** | | | |
| --- | --- | --- | --- |
| **Symbol** | **Gene** | **Symbol** | **Gene** |
| **RPS27A** | **Ribosomal protein S27a** | **IL6** | **Interleukin 6 (interferon, beta 2)** |
| **ACE** | **Angiotensin I converting enzyme (peptidyl-dipeptidase A) 1** | **IL7** | **Interleukin 7** |
| **ACE2** | **Angiotensin I converting enzyme (peptidyl-dipeptidase A) 2** | **IL8** | **Interleukin 8** |
| **ADAM17** | **A disintegrin and metalloproteinase domain 17 (tumor necrosis factor, alpha, converting enzyme)** | **ITGA5** | **Integrin, alpha 5 (fibronectin receptor, alpha polypeptide)** |
| **AGT** | **Angiotensinogen (serine (or cysteine) proteinase inhibitor, clade A (alpha-1 antiproteinase, antitrypsin), member 8)** | **ITGAV** | **Integrin, alpha V (vitronectin receptor, alpha polypeptide, antigen CD51)** |
| **AGTR1** | **Angiotensin II receptor, type 1** | **ITGB1** | **Integrin, beta 1 (fibronectin receptor, beta polypeptide, antigen CD29 includes MDF2, MSK12)** |
| **AGTR2** | **Angiotensin II receptor, type 2** | **ITGB3** | **Integrin, beta 3 (platelet glycoprotein IIIa, antigen CD61)** |
| **ALOX5** | **Arachidonate 5-lipoxygenase** | **KDR** | **Kinase insert domain receptor (a type III receptor tyrosine kinase)** |
| **ANGPT1** | **Angiopoietin 1** | **KIT** | **V-kit Hardy-Zuckerman 4 feline sarcoma viral oncogene homolog** |
| **ANGPT2** | **Angiopoietin 2** | **KLK3** | **Kallikrein 3, (prostate specific antigen)** |
| **ANGPTL3** | **Angiopoietin-like 3** | **MAS1** | **MAS1 oncogene** |
| **ANXA5** | **Annexin A5** | **MMP1** | **Matrix metalloproteinase 1 (interstitial collagenase)** |
| **AZU1** | **Azurocidin 1 (cationic antimicrobial protein 37)** | **MMP14** | **Matrix metalloproteinase 14 (membrane-inserted)** |
| **BAX** | **BCL2-associated X protein** | **MMP2** | **Matrix metalloproteinase 2 (gelatinase A, 72kDa gelatinase, 72kDa type IV collagenase)** |
| **BCL2** | **B-cell CLL/lymphoma 2** | **MMP9** | **Matrix metalloproteinase 9 (gelatinase B, 92kDa gelatinase, 92kDa type IV collagenase)** |
| **BCL2A1** | **BCL2-related protein A1** | **iNOS** | **Nitric oxide synthase 2A / inducible nitric oxide synthase** |
| **BCL2L1** | **BCL2-like 1** | **eNOS** | **Nitric oxide synthase 3 / endothelial nitric oxide synthase** |
| **BLR1** | **Burkitt lymphoma receptor 1, GTP binding protein (chemokine (C-X-C motif) receptor 5)** | **NPPB** | **Natriuretic peptide precursor B** |
| **CASP1** | **Caspase 1, apoptosis-related cysteine protease (interleukin 1, beta, convertase)** | **NPR1** | **Natriuretic peptide receptor A/guanylate cyclase A (atrionatriuretic peptide receptor A)** |
| **CASP10** | **Caspase 10, apoptosis-related cysteine protease** | **OCLN** | **Occludin** |
| **CASP3** | **Caspase 3, apoptosis-related cysteine protease** | **PDGFRA** | **Platelet-derived growth factor receptor, alpha polypeptide** |
| **CASP6** | **Caspase 6, apoptosis-related cysteine protease** | **PDGFRB** | **Platelet-derived growth factor receptor, beta polypeptide** |
| **CCL2** | **Chemokine (C-C motif) ligand 2** | **PECAM1** | **Platelet/endothelial cell adhesion molecule (CD31 antigen)** |
| **CCL20** | **Chemokine (C-C motif) ligand 20** | **PF4** | **Platelet factor 4 (chemokine (C-X-C motif) ligand 4)** |
| **CCL5** | **Chemokine (C-C motif) ligand 5** | **PGF** | **Placental growth factor, vascular endothelial growth factor-related protein** |
| **CDH5** | **Cadherin 5, type 2, VE-cadherin (vascular epithelium)** | **PLA2G4C** | **Phospholipase A2, group IVC (cytosolic, calcium-independent)** |
| **CFLAR** | **CASP8 and FADD-like apoptosis regulator** | **PLAT** | **Plasminogen activator, tissue** |
| **CHGA** | **Chromogranin A (parathyroid secretory protein 1)** | **PLAU** | **Plasminogen activator, urokinase** |
| **COL18A1** | **Collagen, type XVIII, alpha 1** | **PLG** | **Plasminogen** |
| **CPB2** | **Carboxypeptidase B2 (plasma, carboxypeptidase U)** | **PTGIS** | **Prostaglandin I2 (prostacyclin) synthase** |
| **CRADD** | **CASP2 and RIPK1 domain containing adaptor with death domain** | **PTGS2** | **Prostaglandin-endoperoxide synthase 2 (prostaglandin G/H synthase and cyclooxygenase)** |
| **CSF2** | **Colony stimulating factor 2 (granulocyte-macrophage)** | **RHOB** | **Ras homolog gene family, member B** |
| **CSF3** | **Colony stimulating factor 3 (granulocyte)** | **RIPK1** | **Receptor (TNFRSF)-interacting serine-threonine kinase 1** |
| **CX3CL1** | **Chemokine (C-X3-C motif) ligand 1** | **SELE** | **Selectin E (endothelial adhesion molecule 1)** |
| **ECGF1** | **Endothelial cell growth factor 1 (platelet-derived)** | **SELL** | **Selectin L (lymphocyte adhesion molecule 1)** |
| **EDN1** | **Endothelin 1** | **SELPLG** | **Selectin P ligand** |
| **EDN2** | **Endothelin 2** | **SERPINE1** | **Serine (or cysteine) proteinase inhibitor, clade E (nexin, plasminogen activator inhibitor type 1), member 1** |
| **EDN3** | **Endothelin 3** | **SOD1** | **Superoxide dismutase 1, soluble (amyotrophic lateral sclerosis 1 (adult))** |
| **EDNRA** | **Endothelin receptor type A** | **SPHK1** | **Sphingosine kinase 1** |
| **EDNRB** | **Endothelin receptor type B** | **TEK** | **TEK tyrosine kinase, endothelial (venous malformations, multiple cutaneous and mucosal)** |
| **ENPEP** | **Glutamyl aminopeptidase (aminopeptidase A)** | **TFPI** | **Tissue factor pathway inhibitor (lipoprotein-associated coagulation inhibitor)** |
| **F3** | **Coagulation factor III (thromboplastin, tissue factor)** | **TFPI2** | **Tissue factor pathway inhibitor 2** |
| **FGF1** | **Fibroblast growth factor 1 (acidic)** | **THBD** | **Thrombomodulin** |
| **FGF2** | **Fibroblast growth factor 2 (basic)** | **THBS1** | **Thrombospondin 1** |
| **FLT1** | **Fms-related tyrosine kinase 1 (vascular endothelial growth**  **factor/vascular permeability factor receptor)** | **TIMP1** | **Tissue inhibitor of metalloproteinase 1 (erythroid potentiating activity, collagenase inhibitor)** |
| **FLT3** | **Fms-related tyrosine kinase 3** | **TNF** | **Tumor necrosis factor (TNF superfamily, member 2)** |
| **FLT4** | **Fms-related tyrosine kinase 4** | **TNFAIP3** | **Tumor necrosis factor, alpha-induced protein 3** |
| **FN1** | **Fibronectin 1** | **TNFRSF10C** | **Tumor necrosis factor receptor superfamily, member 10c, decoy without an intracellular domain** |
| **ICAM1** | **Intercellular adhesion molecule 1 (CD54), human rhinovirus receptor** | **TNFRSF10D** | **Tumor necrosis factor receptor superfamily, member 10d, decoy with truncated death domain** |
| **ICAM2** | **Intercellular adhesion molecule 2** | **TNFRSF11B** | **Tumor necrosis factor receptor superfamily, member 11b (osteoprotegerin)** |
| **ICAM3** | **Intercellular adhesion molecule 3** | **FAS** | **Fas (TNF receptor superfamily, member 6)** |
| **IFNB1** | **Interferon, beta 1, fibroblast** | **TNFSF10** | **Tumor necrosis factor (ligand) superfamily, member 10** |
| **IL11** | **Interleukin 11** | **FASLG** | **Fas ligand (TNF superfamily, member 6)** |
| **IL14** | **Taxilin** | **VCAM1** | **Vascular cell adhesion molecule 1** |
| **IL15** | **Interleukin 15** | **VEGF** | **Vascular endothelial growth factor** |
| **IL1B** | **Interleukin 1, beta** | **VWF** | **Von Willebrand factor** |
| **IL3** | **Interleukin 3 (colony-stimulating factor, multiple)** | **XDH** | **Xanthine dehydrogenase** |
